# The Value of Optical Coherence Tomography Angiography (OCT-A) in Neurological Diseases

**DOI:** 10.3390/diagnostics12020468

**Published:** 2022-02-11

**Authors:** Albert J. Augustin, Jenny Atorf

**Affiliations:** Staedtisches Klinikum Karlsruhe, Augenklinik, Moltkestr. 90, 76133 Karlsruhe, Germany; jenny.atorf@gmail.com

**Keywords:** optical coherence tomography angiography, retina, neurological disease, microvasculature, central nervous system, biomarker

## Abstract

Optical coherence tomography angiography (OCT-A) was commercially introduced in 2014. OCT-A allows a fast, non-invasive, three-dimensional analysis of the retinal vasculature from the vitreoretinal interface to the choriocapillaris. The results can be evaluated separately in automated or custom-defined retinal layers. Since its introduction, OCT-A has also been used in patients with neurological diseases in order to find and characterize retinal biomarkers. Many neurological diseases have retinal manifestations, often preceding the key symptoms of the neurological disease. Anatomically and developmentally, the retina is a part of the brain. In contrast to the brain, the retina is easily accessible for imaging methods; moreover, retinal imaging is more cost-effective than brain imaging. In this review, the current knowledge about OCT-A findings and possible OCT-A biomarkers in neurological diseases is summarized and discussed regarding the value of OCT-A as a diagnostic tool in neurological diseases.

## 1. Introduction

Per definition, neurological diseases are disorders that affect the brain, the spinal cord and the nerves but also the blood vessels of the nervous system. The group of these diseases is very heterogeneous in terms of their symptoms, causes and progression. Neurological diseases can have genetic as well as environmental causes. Table 1 lists some of the most important neurological diseases that will be covered in this review. The diseases are loosely grouped by their clinical and socioeconomic importance and by the availability of OCT-A data on their retinal manifestation.

Anatomically and developmentally, the retina is a part of the central nervous system (CNS) and thus shares many physiological characteristics with the brain [1]. This includes, among others, the retinal ganglion cells (RGCs) with their typical properties of CNS neurons, or the eye, being an immune-privileged site with, for example, the blood–retina barrier (BRB) that strongly resembles the blood–brain barrier (BBB). Consequently, ocular and especially retinal manifestations of CNS disorders are quite likely and have already been described. Interestingly, ocular manifestations often precede the key symptoms of the CNS disorder, making ophthalmic examinations an important measure for an early diagnosis of those disorders [1]. Additionally, imaging of the retina is much easier and more cost-effective than the available CNS-based imaging methods, a reason why retinal biomarkers could become even more interesting for the diagnosis of neurological diseases [2].

In the late 1990s, the introduction of optical coherence tomography (OCT) revolutionized the objective examination of the retina. OCT is a fast, non-invasive method to analyze both the anatomy of the optic nerve head and of all retinal layers in the macular region [3]. With regard to neurological diseases, particularly the retinal nerve fiber layer (RNFL)—the layer containing the axons of the RGCs—and the RGC layer itself show measurable changes in association with neurological diseases, predominantly thinning of the RNFL and/or RGC layer [4,5].

**Figure 1:**
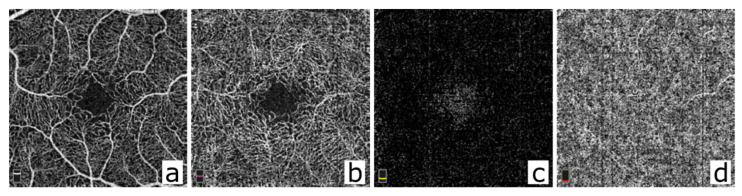
Segmented OCT-A 3 × 3 mm volume scan (304 × 304 A-lines) of a healthy central retina (macula). (**a**) OCT-A scan of the normal superficial capillary plexus located in the ganglion cell layer. The foveal avascular zone (FAZ) is spared by the ramifying vessels. (**b**) OCT-a scan of the normal deep capillary plexus located between the inner nuclear layer and the outer plexiform layer. The vessels form a complex, regular network, and the FAZ is spared. (**c**) OCT-A scan of the normal outer retina. The outer retina is avascular in the healthy eye. (**d**) OCT-A scan of the normal choriocapillaris showing a fine, granular texture. (Scans obtained with Optovue Angio Vue with OCT scanning speed of 70.000 A-scans per second).

Optical coherence tomography angiography (OCT-A) was commercially introduced in 2014. The OCT-A technique offers injection-free, capillary-resolution, and 3-dimensional angiography of the retinal and choroidal vessels [3]. Since its introduction, the OCT-A technique has gained increasing importance in clinical use, as it is a fast and non-invasive method for analyzing retinal and choroidal microvascular structures in a wide variety of diseases. Previously, fluorescence angiography (FLA)—requiring the intravenous injection of fluorescent dyes—was the only possibility to obtain 2-dimensional images of the retinal vasculature. Unfortunately, the interpretation of OCT-A images differs substantially from the interpretation of FLA images. Hence, every clinician has to acquire new interpretation skills to correctly judge the vascular condition of the retina and choroid depicted on OCT-A images (Figure 1).

OCT-A images are obtained by measuring changes in the OCT signal over time. By comparing subsequent OCT scans, the flow signal, which is based on the movement of blood cells, is derived. OCT-A provides 3-dimensional information from up to 4 separate layers of the retina (superficial retinal plexus, deep retinal plexus, avascular outer retina and choriocapillaris) [6]. Many OCT-A systems offer basic quantification tools and a two-visit comparison to visualize changes in blood flow. It is very likely that more quantification tools and more advanced automated analysis tools will be developed and provided by manufacturers in the near future.

**Figure 2:**
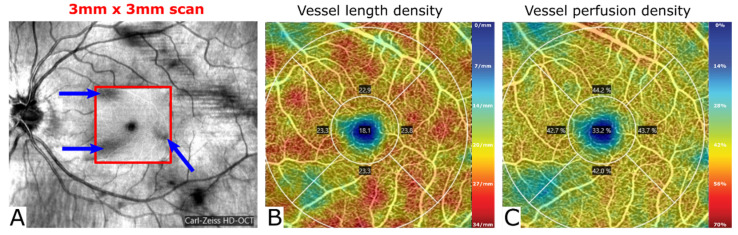
Example of color-coded images of vessel length density and vessel perfusion density of a 3 × 3 mm OCT-A scan of a left eye performed with Zeiss Cirrus 6000 (100.000 scans per second). (**A**) Corresponding fundus image with the location of 3 × 3 mm scan in the center of the macula (red outlined square). Note that the subject has vitreous floaters (blue arrows) inside the scan area that negatively influence the OCT-A signal quality and analysis in the areas below the floaters (shadow artifact). (**B**) Color-coded image of the vessel length density [mm^−^^1^] (Zeiss definition: total length of vessels per unit area; large and small vessels have equal influence on the result). Dark blue areas as the FAZ are completely avascular, red areas have a high vessel length density. Due to low signal quality in the areas below the vitreous floaters, vessel length density is falsely reduced in these areas due to the shadow artifact. Numbers in the sectors of the superimposed EDTRS-grid show the mean sector vessel length density. (**C**) Color-coded image of the vessel perfusion density [%] (Zeiss definition: total vessel area per unit area; large vessels have greater influence than small vessels). Dark blue areas, such as the FAZ, are avascular, and yellow and red areas have higher vessel perfusion density. Due to low signal quality in the areas below the vitreous floaters, vessel perfusion density is falsely reduced in these areas due to the shadow artifact. Numbers in the sectors of the superimposed EDTRS-grid show the mean sector vessel perfusion density. FAZ = foveal avascular zone.

Table 2 gives an overview of commonly used OCT-A parameters and expressions that will occur throughout the text. The default preset automated analysis of OCT-A recordings usually shows vessel (length) density and/or perfusion density results, often in the form of color-coded images overlayed on the infrared fundus picture (Figure 2). Note that vessel (length) density is defined differently between manufacturers (length-based versus area-based measurement; see Figure 2 and Table 2 for details). Frequently used synonyms for vessel density are vascular density, capillary density, flow density and microvascular density.

Many OCT-A systems offer further analyses, such as FAZ metrics (area, diameter, circularity index). Other parameters, for example fractal dimension and lacunarity, are custom-designed analyses that are performed on post-processed binarized OCT-A images [7].

## 2. Method

This review aims at summarizing currently available OCT-A data of patients with neurological diseases and to discuss the value of this fast, non-invasive and cost-effective imaging method for this group of diseases. Literature was searched and obtained from the PubMed database with keyword combinations of ‘optical coherence tomography angiography + x’. X was substituted by one of the following terms: neurological disease/disorder, neuroophthalmology, Alzheimer’s disease, Parkinson’s disease, multiple sclerosis, glaucoma, anterior ischemic optic neuropathy, papilloedema, migraine, stroke, diabetic retinopathy, epilepsy, Leber’s hereditary optic neuropathy, amyotrophic lateral sclerosis, wolfram syndrome, susac syndrome and CADASIL (cerebral autosomal dominant arteriopathy with subcortical infarcts and leukoencephalopathy), respectively. We selected literature based on relevance: retrospective and/or prospective trials that examined OCT-A recordings in neurologic patients. Relevant review articles were also included.

## 3. OCT-A Findings in Neurological Diseases

### 3.1. OCT-A in Alzheimer’s Disease (AD)

AD is the most common neurodegenerative disease in the elderly population and causes significant cognitive impairment. The visual symptoms are loss of contrast sensitivity, color discrimination deficits, difficulties with depth and motion perception, altered pupillary light response, circadian rhythm disturbances and sleeping disorders. Mirzaei et al. recently reviewed the pathophysiology of AD and mild cognitive impairment (MCI), with a special focus on the ophthalmic manifestation [8]. In short, the pathophysiology of AD in the brain is characterized by the accumulation of ß-amyloid, tauopathy as well as cerebral amyloid angiopathy and a neuroinflammatory component [8]. In 2010, the group of Koronyo–Hamaoui identified the existence of ß-amyloid accumulation in the retina [9]. One year later, the group of Schön et al. first described tau hyperphosphorylation (pTau) in the retina, the second hallmark of AD [10]. These findings underline the striking similarities between the retinal and cerebral phenotypes of AD, leading to the assumption that pathophysiological changes in the retina and their anatomical and physiological consequences could be a potent biomarker for AD. Direct in-vivo imaging of ß-amyloid or pTau in the retina is under investigation [10].

For the OCT-A-based detection of microvascular changes in the AD retina, cerebral amyloid angiopathy (CAA) is of particular interest. CAA has been described in the brain of AD patients and is characterized by the deposition of amyloid in the walls of cerebral arteries, which triggers several ischemia-induced pathogenic molecular pathways, finally leading to hemorrhagic complications [8,11]. CAA has been reported to be present in the retina, yet it is unclear if it occurs to the same extent as in the brain. So far, several retinal vascular dysfunctions have been reported in AD, such as increased tortuosity, narrowed veins, decreased blood flow, compromised branching complexity and microvascular network damage [8].

**Figure 3:**
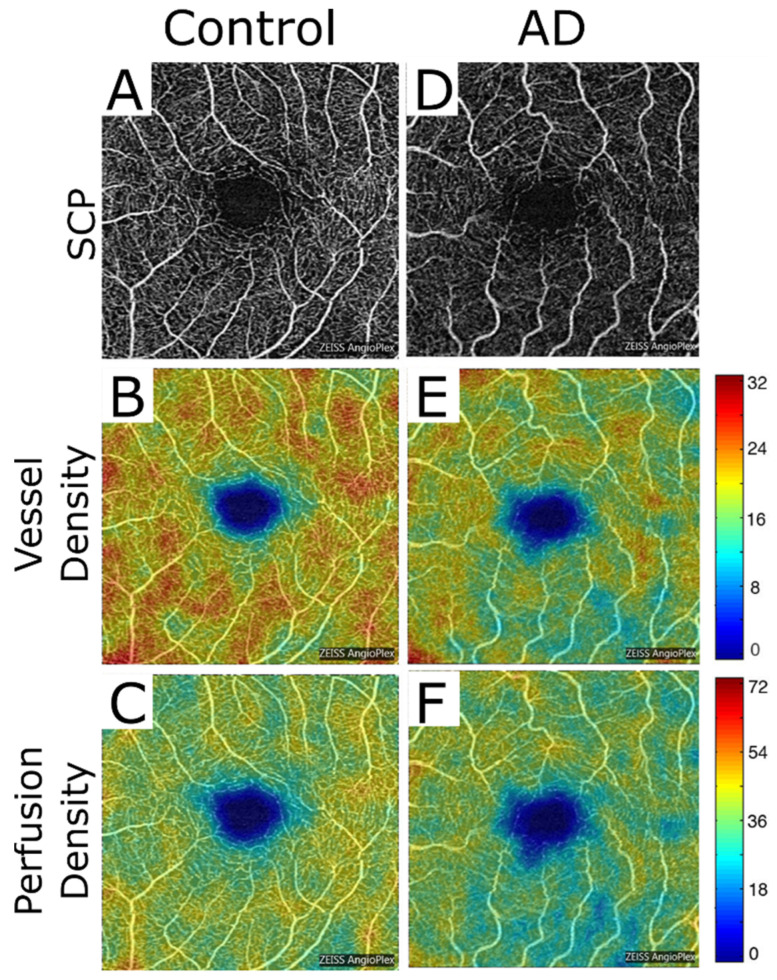
Macular OCT-A scans sized 3x3mm (68.000 A-scans per second) of the superficial capillary plexus of a healthy subject (**A**–**C**) and of a patient with AD (**D**–**F**). The vessel density (**B**,**E**) and the perfusion density (**C**,**F**) is reduced in the AD patient. (reprinted with permission from ref. [15]. Copyright 2019 Yoon et al.; scans obtained with Zeiss Cirrus 5000).

Three recent review articles have summarized OCT-A findings in AD patients [11,12,13]. Essentially, OCT-A examinations of AD retinas have shown an enlarged FAZ, a decrease of the vascular density of the superficial and deep vascular plexus in the macular region (Figure 3), and also in the radial peripapillary capillary layer. One group specifically analyzed the retinas of preclinical AD patients and reported capillary dropout areas in the fovea, leading to an enlargement of the FAZ [14]. The latter indicates that preclinical AD patients already have measurable changes in the retinal vasculature. When compared with patients with mild cognitive impairment (MCI), which is considered to be an intermediate stage between normal aging and dementia, AD patients have more severe reductions of the retinal microvascular density [15]. Whether there is a correlation between disease severity (i.e., duration of disease or time since initial diagnosis) and changes in retinal vessel density in AD patients has not yet been investigated and should be addressed by future trials. For conventional OCT analyses in AD patients, clear correlations with disease severity have been reported for the reduction of GCL and RNFL thickness [16].

### 3.2. Parkinson’s Disease (PD)

PD is the second most common neurodegenerative disease after AD. PD is related to the progressive loss of dopaminergic neurons as a consequence of α-synuclein accumulation within neuronal cytoplasm [7]. As a result, PD manifests with motor symptoms such as bradykinesia, resting tremor and rigidity [17,18]. Moreover, non-motor symptoms are also described, including sleep disorders, anosmia, depression, cognitive impairment and vision deficits [17].

Several groups have investigated OCT-A changes in the retinas of PD patients. In 2020, Zou et al. published results of combined OCT and OCT-A imaging in PD patients and healthy subjects [17]. The authors quantified the FAZ and the superficial retinal vessels with OCT-A and used conventional OCT to obtain macular parameters (central macular thickness (CMT), macular retinal thickness (MRT), total macular volume (TMV) and average ganglion cell-inner plexiform layer complex (GCL-IPL)) and the peripapillary RNFL thickness (overall, temporal, nasal, inferior and superior) [17]. Regarding the OCT-A results, PD patients had significantly lower vessel length density (VLD), reduced vessel perfusion density (VPD) and a reduced FAZ circularity index but the same FAZ area size on the macular OCT-A scans in comparison to healthy subjects. Zou et al. further explored that the OCT-plus-OCT-A combination is a better biomarker for both the diagnosis and for the detection of PD progression as compared to either technique alone [17].

The group of Rascunà et al. performed a similar comparative study to elucidate a possible correlation between retinal thickness (measured with OCT) and retinal microvasculature (measured with OCT-A) [18]. They found that the peripapillary and macular RNFL, GCL, IPL, and INL were thinner in PD patients. Moreover, there was a positive correlation between RNFL, GCL and IPL thickness, and microvascular density in the foveal region [18]. As these findings were already present at early stages of the disease and were independent of dopaminergic treatment, the authors concluded that the retina could be a valuable biomarker for PD diagnosis and progression evaluation.

Robbins et al. characterized retinal microvascular and choroidal structural changes in PD patients and healthy subjects using macular OCT-A scans sized 6 × 6 mm. They found significantly decreased retinal vessel density and perfusion density in the superficial capillary plexus [19]. In a second study, Robbins et al. reported significantly higher capillary perfusion density and capillary flux index in the 4.5 mm × 4.5 mm peripapillary region of PD patients [20]. The authors concluded that OCT-A could be a noninvasive method to identify novel biomarkers for the early detection of PD.

The group of Murueta-Goyena et al. recently described the results of their foveal remodeling of the retinal microvasculature [7]. They used 10° × 10° large central OCT-A scans of the fovea and performed various analyses of the superficial and deep capillary plexus in the inner 1 mm circle (fovea) and the parafoveal ring (1 to 2.5 mm). Their analyses included several post-processing steps of the originally obtained angiographic images. Besides the common analysis of the perfusion density and FAZ size and circularity, they also measured parameters such as density of vessel skeleton, vessel perimeter index, mean vessel diameter, fractal dimension (describes shape or texture, and determines complexity of an image), and lacunarity (expresses patchiness or inhomogeneity of an image). Interestingly, they found that the FAZ area was significantly smaller in PD patients, contrasting the findings of Zou et al. Moreover, perfusion density, vessel perimeter, fractal dimension and lacunarity were increased in the foveal 1mm zone in PD patients compared to controls. In the parafoveal ring, lacunarity was significantly decreased in the superficial plexus and significantly increased in the deep plexus. All other parameters were not different. The authors also determined the accuracy of the parameters regarding their diagnostic ability in the detection of PD. Three parameters showed excellent diagnostic accuracy. These were the lacunarity of the deep plexus in the fovea and the lacunarity of the superficial and deep plexus in the parafoveal ring [7]. The authors concluded that lacunarity could become a reliable biomarker for PD diagnosis if OCT-A plexus segmentation and image post-processing becomes more standardized.

None of the above-mentioned studies investigated whether disease severity had an influence on the OCT-A findings. Zou et al. indeed rated the disease severity of the PD patients into 3 stages, but OCT-A findings were not analyzed stage-specifically, probably due to the small number of total patients [17].

### 3.3. Multiple Sclerosis (MS)

**Figure 4:**
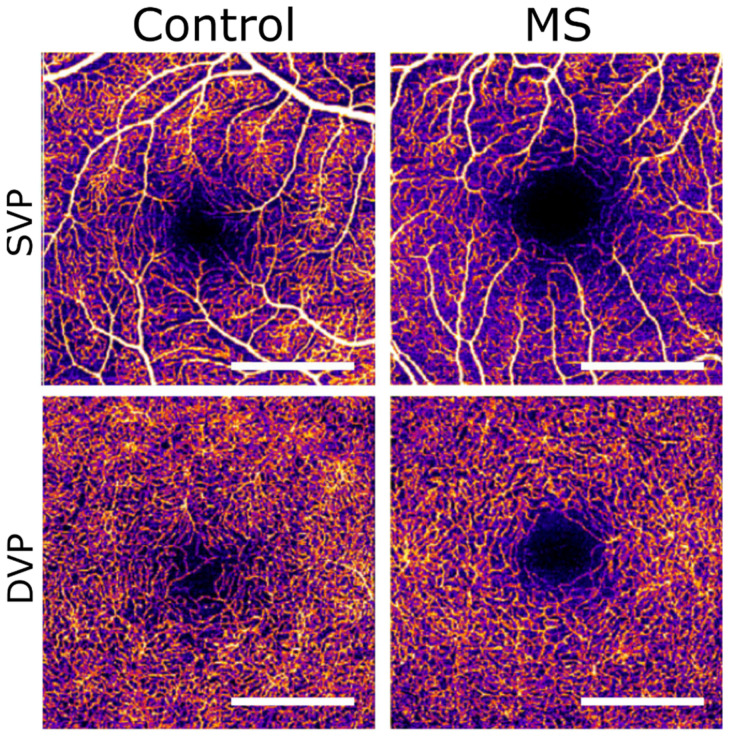
OCT-A scans of superficial (SVP) and deep (DVP) vascular plexus of a healthy control (left column) and of an MS patient (right column). The FAZ is enlarged in the retina of the MS patient. Scale bar: 1mm. Scans are 3 × 3 mm and obtained with the Heidelberg Engineering OCT2 system with 85.000 scanning speed. FAZ = foveal avascular zone, MS = multiple sclerosis. (reprinted with permission from ref. [26]. Copyright 2020 Kleerekooper et al.).

MS is a chronic neurodegenerative disease characterized by demyelination in different CNS regions caused by (autoimmune) inflammatory processes. Ophthalmic findings include blurred or double vision and optic neuritis (ON). ON is the onset symptom in approximately 20% of patients with MS and it occurs in about 50% of patients during the course of the disease. Several studies have investigated OCT-A findings of the foveal avascular zone (FAZ) and macular and peripapillary vessel densities in subjects with MS [21,22,23,24,25]. Yilmaz et al. found no changes in FAZ metrics between MS patients and controls, but significant reductions in vessel density of the superficial and deep capillary plexus in the macular region [21]. The reduction of vessel density was more pronounced in MS patients who had ON. Aly et al. found that FAZ size is increased in MS patients that have a history of ON, but not in MS patients without an ON history (Figure 4) [25]. The group of Farci et al. found significant flow density reductions in the superficial capillary plexus and in the choriocapillaris layer, and partially reduced flow densities in the deep capillary plexus [22]. Cordon et al. reported decreased retinal vascularization density in the superficial capillary plexus of the parafoveal retina [23]. Specifically, the vessel density was reduced in the nasal, superior and inferior parafoveal retina. The authors also found lower vessel densities in patients with a longer history of MS, but no correlations of ON. Finally, Cennamo et al. found reduced vessel densities for the superficial capillary plexus and the peripapillary capillary plexus in MS patients [24]. The vessel density of the deep capillary plexus and of the choriocapillaris was not significantly different from the control group.

To our knowledge, there is no study that aimed to analyze whether there is a correlation of OCT-A changes and disease severity or disease duration. Some groups, however, evaluated single findings in relation to the EDSS score (disability score). Spain et al. exclusively analyzed the OCT-A flow index of the optic nerve head (ONH-FI) and found that MS patients with and without a history of ON had reduced ONH-FI, but it was not related to the disability score [27]. Similarly, Cennamo et al. also found no correlation between OCT-A results and disability score or disease duration [24]. Some other groups, however, reported correlations of OCT-A results with clinical measures in MS patients. ON, for example, is associated with rarefication of the superficial and deep retinal vessels and ongoing inflammatory disease activity was linked to higher choriocapillary vessel density [28]. Jiang et al. reported a positive correlation of the vessel density with disability (EDSS score) and disease duration [29]. On the contrary, Murphy et al. found a correlation of decreased superficial vessel density with higher EDSS scores and lower visual acuity scores [30]. Larger longitudinal trials, ideally in a prospective setting, should clarify whether there is a correlation between OCT-A findings and disease progression and whether the vessel density decreases or increases. The latter could be related to active inflammatory disease stages (relapsing-remitting MS).

### 3.4. Glaucoma

Very recently, the AAO published a review on the use of OCT-A for the diagnosis of primary open-angle glaucoma (POAG) [31]. In summary, OCT-A measurements of the microvasculature in the superficial peripapillary region show that the vessel density is reduced in glaucoma patients when compared with healthy controls. In addition, most of the reviewed studies also confirmed that vessel density loss correlates with glaucoma severity. Further, a subgroup of studies reported moderate to strong correlations with visual field defects or RNFL thickness.

OCT-A of the macular microvasculature is altered in glaucoma patients. The macular vessel density is decreased in moderate and severe glaucoma eyes and most investigations showed correlations with the ganglion cell complex (GCC) and/or visual field losses.

Comparisons of the FAZ in glaucomatous and healthy eyes revealed that glaucomatous eyes have an increased FAZ diameter and a reduced FAZ circularity index. Interestingly, the FAZ circularity index was found to be highly accurate for the discrimination between healthy and glaucomatous eyes. Nevertheless, the authors state that overall, the peripapillary OCT-A measurements showed better discrimination ability than macular OCT-A.

In conclusion, OCT-A, especially that of the ONH, provides useful complementary information for the diagnosis or about the progression of the disease. In particular, OCT-A analyses have the potential to evaluate and identify glaucoma suspects [31].

### 3.5. Anterior Ischemic Optic Neuropathy (AION)

AION is a consequence of decreased blood supply of the anterior optic disc by diseased small vessels [32]. This neuropathy can be divided into an arteritic (AAION) and non-arteritic (NAION) type, and approximately 85% of the cases are non-arteritic. AAION has an acute inflammatory component within the lumen of the artery, with giant cell arteritis being the most common cause, whereas NAION is associated with cardiovascular risk factors [2].

Several groups reported OCT-A measurements of optic nerve head perfusion of AAION or NAION patients in the acute or non-acute state [32,33,34,35,36,37]. In summary, non-acute NAION shows significantly increased optic disc non-perfusion areas that correlate well with visual field defects and best corrected visual acuity (BCVA) [32,34]. In the acute phase of either AAION or NAION, the edema of the disc may obscure the peripapillary choriocapillaris or lead to artifacts, but dilation and increased tortuosity of the vessels can be seen in the superficial capillary plexus [34,36,37]. Rougier et al. specifically examined acute papilloedema of different causes (NAION, papillitis and papilloedema) and found that OCT-A is mainly able to differentiate between the types using morphological rather than quantitative perfusion changes [37]. Similarly, the group of Pierro et al. described vessel density and vessel tortuosity changes in acute AAION and NAION cases that correlated well with RNFL reduction and visual field defects [36]. A case report study of a chronic AAION explicitly measured OCT-A of the macular region and found that only the superficial capillary plexus was decreased, whereas deep capillary plexus and choriocapillaris were normal [35].

### 3.6. Papilloedema

The term papilloedema is used to describe swelling of the optic disc in association with elevated intracranial pressure. Papilloedema usually manifests bilaterally, and visual function is generally preserved. With these characteristics, papilloedema is easily distinguishable from other diseases with optic disc swelling, i.e., AION or papillitis [38]. In addition to the latter, papilloedema has to be distinguished from pseudopapilloedema, which describes a congenital elevation of the optic disc with similar ophthalmoscopic features as papilloedema. With conventional fluorescein angiography, papilloedema is clearly distinguishable from pseudopapilloedema. Fard et al. examined whether OCT-A has the same potential to differentiate between the two. Using custom image analysis software, the group found that the peripapillary capillary vessel density was significantly smaller in pseudopapilloedema than in papilloedema, especially in the nasal quadrant [39]. Rougier et al. compared OCT-A findings of eyes with NAION, papillitis and papilloedema [37]. They found that eyes with papilloedema had dilated and tortuous capillaries at the optic disc surface, but the peripapillary vessel network showed no changes. In comparison with acute optic disc swelling in AION and papillitis, the authors stated that morphologic vessel changes were able to differentiate between the three kinds of optic disc edema [37]. A recent retrospective study by Chonsui et al. reported that eyes with papilloedema secondary to increased intracranial pressure have reduced peripapillary capillary vessel density but normal capillary flux indices when compared to healthy eyes. [40].

### 3.7. Migraine

Migraine is a common neurological disorder with a strong genetic component and the involvement of several brainstem regions. It is highly prevalent, affecting approximately 12% of the population. Women are affected more often than men [41]. The pathophysiology of migraine is complex, and vasculature changes are partially involved [42]. About one-third of migraine patients experience a visual manifestation of the disease in the form of a migraine aura. Migraine auras are reversible visual disturbances that can last from 5 to 60 min and that precede the headache attack [43].

OCT-A analyses in patients with migraine are not consistent. Most groups report no significant changes in macular and/or peripapillary vessel perfusion [42,44,45,46,47], while some authors found significant changes, interestingly more pronounced in migraine patients with aura than without [44,48]. In addition, one case report suggests that vascular changes are transient during the aura attack and resolve some hours afterwards [48]. Two publications found some weak negative correlations between disease duration and central choroidal thickness and parafoveal superficial capillary plexus vessel density, respectively [46,47].

### 3.8. Stroke

Stroke is the second leading cause of death and disability in the elderly population worldwide [49], and more than 80% are of the ischemic type versus 20% of the hemorrhagic type [50]. Stroke is associated with several modifiable and non-modifiable risk factors. Nonmodifiable risk factors are age, sex, genetics, ethnicity and transient ischemic attack (TIA). Modifiable risk factors are hypertension, smoking, alcohol and drug abuse, diet, physical inactivity, hyperlipidemia, and diabetes mellitus [51]. During the acute phase of a stroke, the blood supply to a part of the brain is reduced or interrupted. Conventionally, cerebral vasculature is evaluated by invasive digital subtractive angiography (DSA), magnetic resonance imaging (MRI) or by computed tomography (CT). OCT-A cannot replace the aforementioned methods for the acute diagnosis of stroke events, i.e., detection of the stroke site. However, the association between retinal vascular changes and cerebrovascular diseases (CVD), such as stroke, is well known and has recently been summarized in a review by Rim et al. [52]. Monitoring of retinal vascular changes, for example with OCT-A, could identify patients at high risk for CVD in general or patients at risk for recurrent CVD events [52]. Additionally, patients who have been diagnosed with branch retinal artery occlusion (BRAO), central retinal artery occlusion (CRAO) or ocular ischemic syndrome (OIS) are at higher risk of developing strokes, as reported by a retrospective study from Avery et al. [53].

OCT-A has been used to characterize stroke-induced perfusion changes in the retina. Liu et al. characterized retinal microvasculature changes using OCT-A and found that vessel density was reduced in the superficial and deep capillary plexus as well as in the peripapillary region compared to controls [54]. They also found differences in several FAZ parameters, such as area, circularity, and perimeter. The regression analyses confirmed that OCT-A was able to distinguish stroke patients from controls with good sensitivity and specificity. Molero-Senosiain et al. performed OCT-A in stroke patients with ischemic lesions affecting the visual pathway and in a healthy control group [55]. All included stroke patients had partial visual field defects as a consequence of the ischemic attack. It is postulated that retrograde trans-synaptic degeneration of ganglion cells is the underlying mechanism that leads to visual field defects. Compared to the control group, the vessel density was reduced in all vascular plexuses in the macular and peripapillary regions. The authors do not report more pronounced microvascular changes in areas corresponding to the visual field defects, but the thickness of the GCL correlated with the visual field defects [55].

In conclusion, OCT-A could become a valuable method for monitoring retinal vascular changes in patients who have suffered a stroke. OCT-A cannot replace conventional methods used in the acute phase of stroke diagnosis. However, it could provide useful information about retinal microvascular changes in former stroke patients to identify patients at high risk for recurrent stroke events. Similarly, BRAO and CRAO patients could be regularly monitored with OCT-A examinations to detect worsening of the retinal microvascular perfusion.

### 3.9. Diabetic Neuropathy (Neurodegenerative Component)

Diabetic retinopathy (DR) is a multifactorial complication of diabetes mellitus (DM) [56], and it becomes more and more evident that DR is not only a microvascular complication but also has a neurodegenerative component besides its inflammatory nature. The risk of DR increases with the duration of DM. The earliest changes in the retina are microaneurysms and capillary dropout. With progression of the disease, proliferative changes, diabetic macular edema (DME), vitreomacular changes and vitreous bleeding can occur.

Some years ago, different investigations suggested that especially DM type 2 shares similarities with the pathophysiologic pathways of Alzheimer’s disease [57,58,59,60]. Further, it seems that this neurodegenerative component manifests early in the course of DR [59]. Retinal neurodegeneration is associated with low-grade inflammation, immune cell activation, extracellular glutamate accumulation and an imbalance of the production of neurotrophic factors. As a consequence, glial activation and neuron apoptosis are induced [59]. These physiological changes lead to several functional, vision-related consequences, such as decreased contrast sensitivity, decreased hue discrimination, delayed dark adaptation, reduced visual field sensitivity and decreased visual acuity [59].

It is postulated that type 2 DM patients who show this early neurodegenerative component are at higher risk of developing Alzheimer’s disease. Methods for the early detection of the associated retinal vascular changes could be highly helpful for the diagnosis and prognosis of this disease component. Additionally, it provides the opportunity for the application of early, individualized therapeutic strategies.

Several review articles have summarized early microvascular changes in the diabetic retina that can be detected with OCT-A [61,62,63]. Zhang et al. even focused on microvascular changes that are present before any clinical signs of DR [63]. In summary, the retinas of DM patients without DR had enlarged FAZ areas, decreased perfusion density in both the superficial and deep vascular plexus of the macula, and reduced radial peripapillary vessel density [63].

### 3.10. Epilepsy

It is well known that conventional OCT shows significant reductions of the GCL, RNFL and central retinal thickness in patients with epilepsy [64]. Moreover, RNFL thinning showed a negative correlation with the duration of antiepileptic drug use [65].

In 2002, the group of Hilton et al. reported reduced neuroretinal blood flow, volume and velocity measured by scanning laser Doppler flowmetry in epileptic patients taking antiepileptic drugs [66].

Until today, OCT-A-based characterization of the retinal vasculature in epileptic patients is still missing, although changes of the cerebral and retinal blood flow are known to occur in these patients. It is probable that OCT-A could detect alterations of the retinal microvasculature and it is desirable to elucidate these alterations in the future.

### 3.11. Leber’s Hereditary Optic Neuropathy (LHON)

LHON is a rare hereditary disease that is caused by certain point mutations in the mitochondrial DNA. Remarkably, LHON is highly tissue restrictive, as it only manifests in the RGCs and their axons [67]. The predominantly male patients with LHON typically lose vision in their second or third decade of life. In its acute phase, visual acuity progressively declines from 100% to 10% or less. In the retina, the sub-acute phase is characterized by optic disc hyperemia, pseudoedema, abnormalities of the peripapillary blood vessels and swelling of the RNFL. Later, in its chronic atrophic phase, optic disc pallor is seen as a consequence of RGC loss [68]. OCT-A measurements are available, mostly from case reports, of the chronic phase and fewer from the sub-acute phase of the disease [68,69,70]. The greatest patient group was analyzed by Yu et al. [67]. Already in the sub-acute phase, the radial peripapillary capillary (RPC) vessel density is significantly lower in the temporal and inferior sectors than in control eyes. Similarly, the parafoveal superficial capillary plexus vessel density is decreased. In the chronic phase, the RPC vessel density is reduced in all sectors, and it is significantly smaller than in the sub-acute phase. The parafoveal vessel density did not decline significantly lower than in the sub-acute phase [67]. Further, the reduction of the RPC vessel density correlates well with the RNFL thickness reduction. Kousal et al. reported that microvascular dropout was restricted to the RNFL and GCL, an important finding in differentiating between other diseases with optic neuropathy and vessel density changes [68].

### 3.12. Amyotrophic Lateral Sclerosis (ALS)

ALS is a progressive neurodegenerative disease that affects the upper and lower motor neurons, leading to paralysis and consequent respiratory failure within 3–5 years after diagnosis [71]. Nowadays, evidence is increasing that ALS is a very complex disease with several possible non-motor symptoms that have been under-evaluated in former trials [72]. One of the non-motor neuron related symptoms include changes of small capillaries [73]. The presence of retinal manifestations in general has been reviewed by Cervéro et al. [73]. To date, there are reports about changes in conventional OCT parameters in ALS patients: (a) reduction of the total macular thickness, (b) thinning of RNFL and INL, but not GC-IPL, and (c) reduction of ONL thickness (reported by one study). Some results indicate that both eyes of the same patient may be affected asymmetrically [71,73]. Analyses about retinal vasculature using OCT-A are missing at this time and should be performed in the future.

### 3.13. Wolfram Syndrome

Wolfram syndrome is a rare genetic, progressive disorder with a poor prognosis (median age at death: 30 years). It is characterized by juvenile-onset diabetes mellitus, diabetes insipidus, neurodegeneration, hearing loss and optic nerve atrophy [74]. The optic nerve atrophy leads to gradual visual acuity loss, color discrimination deficits and visual field defects [75]. A case report of OCT-A examination of a patient with Wolfram syndrome showed decreased parameters of the peripapillary vascular network that corresponded to the regions of RNFL thinning [75]. These findings were more pronounced in the temporal sector. OCT-A examinations should be performed in a larger number of patients with Wolfram syndrome to investigate whether the disease leads to characteristic OCT-A changes.

### 3.14. Susac Syndrome

Susac sysndrome is a rare, presumably auto-immune-mediated condition resulting in the occlusion of small arteries, most often in the brain, inner ear and retina resulting in encephalopathy, low- to mid-frequency hearing loss and visual disturbances as a consequence of branch retinal artery occlusion (BRAO) [76]. Only 13% of the patients suffer from all three aforementioned features at disease onset, making it a challenge to properly diagnose Susac syndrome.

Ocular findings include BRAO and its consequences. Funduscopically, sectorial whitening may occur (cave: may be transient) as well as yellowish Gass plaques. More rarely, retinal neovascularizations and/or vitreous hemorrhage develop as a consequence of retinal ischemia. More recently, peripheral arterio-arterial collaterals and arteriovenous collaterals have been described [76]. Functionally, visual field loss or central or paracentral scotoma are reported.

OCT-A findings in patients with Susac syndrome are vascular hypoperfusion in the superficial and deep capillary plexus in regions affected by the BRAO [76,77]. Choriocapillaris perfusion was normal [76].

### 3.15. Cerebral Autosomal Dominant Arteriopathy with Subcortical Infarcts and Leukoencephalopathy (CADASIL)

CADASIL is a rare autosomal dominant disease. It is the most common cause of hereditary ischemic strokes [2,78]. Due to a mutation of the Notch-3 gene, the vessel wall of the brain vasculature thickens and lumen stenosis occurs [2].

Alten et al. were the first to describe multimodal imaging of the retinal vessels in CADASIL patients shortly before the commercial introduction of OCT-A. Alten et al. used a confocal laser scanning ophthalmoscope (cSLO), conventional OCT and fluorescein and indocyanine-green angiography [79]. They found arteriovenous nicking and venous dilation in a great proportion of CADASIL eyes. In addition, mean arterial and venous outer diameters as well as vessel wall thickness were significantly increased. Four years later, the group described OCT-A changes in CADASIL patients [80]. They found significantly reduced vessel density in the deep capillary plexus in the macular region. All other macular and optic nerve head OCT-A parameters were not different when compared to the control group.

## 4. Discussion

OCT-A is a fast and non-invasive method to characterize the retinal vasculature in separate layers, from the normally avascular vitreoretinal interface to the superficial and deep capillary plexus to the choroid. The typically described parameters are the vessel density and the capillary flow index both for macular and optic nerve head (ONH) scans. Moreover, the size and shape of the FAZ can be assessed on macular OCT-A images. Some groups have established custom designed post-processing procedures of the obtained images and have described further parameters, such as the fractal dimension (describes shape or texture, and determines complexity of an image) or the lacunarity (expresses patchiness or inhomogeneity of an image) [7]. Additionally, OCT-A images reveal apparent morphological changes, such as tortuosity, micro- and macroaneurysms, and vessel thickening or thinning.

Notably, for the interpretation of OCT-A scans, the physician or otherwise qualified person has to adopt completely new skills, as the interpretation is substantially different from that of conventional fluorescence angiography images.

Since its introduction in 2014, OCT-A has been used with increasing frequency in a wide variety of ophthalmic and non-ophthalmic conditions. With the retina being a part of the brain and hence sharing many anatomical and physiological similarities, it is termed a “window to the brain”. It is well known that many primordial brain disorders have manifestations in the eye, especially in the neurosensory retina. In contrast to the brain, the retina is readily accessible by several imaging modalities, including OCT-A. Above that, retinal imaging is more cost-effective than brain imaging. Not surprisingly, researchers have the intention to find and characterize retinal biomarkers for the early detection, prognosis, progression and/or therapy efficacy evaluation of—not only—neurological diseases.

Neurological diseases, especially those with increasing prevalence due to the aging population, are of special interest as they pose an increasing socioeconomic burden now and in the future.

As described above, OCT-A is a promising candidate for the identification of retinal biomarkers for neurological diseases. Many, if not all, of the neurological diseases described herein have been shown to alter one or more retinal vascular parameters, underlining the potential of OCT-A to be used as a standard diagnostic tool in non-ophthalmic diseases in the near future. The potential of OCT-A to become a standard screening and progression monitoring method is probably even greater.

For that purpose, there are still some limitations that have to be addressed. One major point is, for example, that segmentation of the different layers of automated OCT-A analysis is not consistent between the machines of different manufacturers. Therefore, OCT-A results are not comparable without limitations if performed on different machines. Above that, more standardization is needed for automated as well as customized post-processing analyses of the obtained OCT-A scan data.

Apart from these technical issues, there is a need for larger trials to assess the specificity and sensitivity of the identified biomarker candidates. Furthermore, detailed descriptions of each suitable biomarker are required for each disease. Above that, for all progressive neurological diseases, characterization of OCT-A biomarkers depending on disease severity is missing. Longitudinal trials should be performed to investigate correlations between OCT-A changes and disease severity.

OCT-A examinations of patients with neurological diseases are probably more prone to image artifacts due to mental and/or physical issues (i.e., Alzheimer’s or Parkinson’s patients). These can result in poorer fixation or strong eye movement, which can only be partially ameliorated by inbuilt systems (e.g., eye tracking) that ensure and increase good image quality, such as eye tracking software. In elderly patients, media opacities can additionally reduce the quality of imaging. Most OCT-A machines display the image quality by a numeric value. This value is a good indicator of the image quality and validity of the results during clinical routine. For clinical trials, however, images below a predefined quality minimum have to be excluded from the analysis to minimize interpretation errors due to low image quality.

Clearly, OCT-A can never be the single tool for the diagnosis of a neurological disease. For example, until today, no disease-specific pattern of OCT-A changes has been described that would allow the diagnosis of a neurological disease solely based on an OCT-A examination. As described in the results section, the majority of OCT-A changes in association with neurological diseases are overall reductions of vessel and/or perfusion density that are not specific for a certain disease. Rarely, for example, in MS patients, specific reductions of the vessel density in distinct parafoveal quadrants (nasal, superior and inferior) have been described [23]. Whether this pattern is specific for MS and whether it has the potential to serve as an exclusive diagnostic parameter for MS has to be confirmed in the future. As mentioned earlier, it is inevitable and of great importance to characterize and describe every potential OCT-A biomarker in detail for every neurological disease. In the meantime, OCT-A examinations are currently only able to provide complementary results in addition to key diagnostic procedures that are necessary for the reliable diagnosis of a certain disease. However, in the future, OCT-A can hopefully substitute some cost-effective, invasive procedures to reduce the burden for the patients on one side and to reduce healthcare costs on the other side. For some diseases, OCT-A also has the potential to become an early screening method and to some extent, it may be able to be used to prognose disease progression. Probably, the potential to become an early screening method is much greater than the potential to become a reliable diagnostic tool for neurological diseases, as disease-specific changes seem to be hardly definable.

## Figures and Tables

**Table 1:** Neurological diseases grouped for social and clinical importance. AION = anterior ischemic optic neuropathy, NAION = non-arteritic anterior ischemic optic neuropathy, LHON = Leber’s hereditary optic neuropathy, CADASIL = cerebral autosomal dominant arteriopathy with subcortical infarcts and leukoencephalopathy.

Group 1
Alzheimer’s Disease (AD)
Parkinson’s Disease (PD)
Multiple Sclerosis (MS)
Glaucoma
AION + NAION
Papilloedema
Migraine
Stroke
**Group 2**
Diabetic Retinopathy (DR) (neurodegenerative component)
Epilepsy
**Group 3**
LHON
Amyotrophic lateral sclerosis (ALS)
Wolfram Syndrome
Susac Syndrome
CADASIL

**Table 2:** Commonly used OCT-A expressions and parameters. * Vessel density can be measured by area- or length-based measurements. Length-based measurements are more sensitive to changes of small capillaries; in area-based measurements, larger vessels have greater influence. Synonyms for vessel density: vascular/microvascular/capillary/flow density.

Parameter	Definition
Vessel length density *	Length-based measurement of vessel density: total length of the perfused vasculature per unit area in the region of measurement (Zeiss definition)
Vessel density *	Area-based measurement of vessel density: expresses how much area is taken up by vessels (Optovue definition)
Perfusion density	Area-based measurement of vessel density: total area of the perfused vasculature per unit area in the region of measurement (Zeiss definition)
Fractal dimension	Describes shape or texture and determines complexity of an image
Lacunarity	Expresses patchiness or inhomogeneity of an image
Vessel perimeter index	Expresses vessel perimeter in relation to total image area
Foveal avascular zone (FAZ)	Avascular area in the center of the macula within the fovea
FAZ area	Area of the FAZ
FAZ diameter/perimeter	Diameter/Perimeter of the FAZ
FAZ circularity index	Index describing how circular the area of the FAZ is; values closer to 1 indicate higher circularity
Vessel tortuosity	Abnormal curvature of the vessels
Narrowed/dilated vessels	Morphologically obvious thinning or dilation of vessels
Branching complexity	Altered complexity of vessel branching
